# Prospective monitoring of chronic myeloid leukemia patients from the time of TKI discontinuation: the fate of peripheral blood CD26^+^ leukemia stem cells

**DOI:** 10.3389/fphar.2023.1194712

**Published:** 2023-05-26

**Authors:** Paola Pacelli, Adele Santoni, Anna Sicuranza, Elisabetta Abruzzese, Valentina Giai, Monica Crugnola, Mario Annunziata, Sara Galimberti, Alessandra Iurlo, Luigiana Luciano, Federica Sorà, Carmen Fava, Elena Bestoso, Cristina Marzano, Alessandra Cartocci, Marzia Defina, Vincenzo Sammartano, Emanuele Cencini, Donatella Raspadori, Monica Bocchia

**Affiliations:** ^1^ Hematology Unit, University of Siena, Azienda Ospedaliera Universitaria Senese, Siena, Italy; ^2^ Sant'Eugenio Hospital, Tor Vergata University, Rome, Italy; ^3^ Division of Hematology, Città Della Salute e Della Scienza, Turin, Italy; ^4^ Ematologia e Centro BMT, Azienda Ospedaliero-Universitaria di Parma, Parma, Italy; ^5^ Hematology, Cardarelli Hospital, Naples, Italy; ^6^ Department of Clinical and Experimental Medicine, Section of Hematology, University of Pisa, Pisa, Italy; ^7^ Hematology Division, Foundation IRCCS Ca’ Granda Ospedale Maggiore Policlinico, Milan, Italy; ^8^ Hematology, Department of Clinical Medicine and Surgery, Federico II University, Naples, Italy; ^9^ Sezione di Ematologia, Dipartimento di Scienze Radiologiche ed Ematologiche, Università Cattolica del Sacro Cuore, Rome, Italy; ^10^ Azienda Ospedaliera Ordine Mauriziano di Torino, Turin, Italy; ^11^ Department of Medical Biotechnologies, University of Siena, Siena, Italy

**Keywords:** chronic myelogenous leukemia, treatment free remission, CD26+, leukemia stem cell, TKI, flow cytometry

## Abstract

**Introduction:** In chronic myeloid leukemia (CML), about half of the patients achieving a deep and stable molecular response with tyrosine kinase inhibitors (TKIs) may discontinue TKI treatment without disease recurrence. As such, treatment-free remission (TFR) has become an ambitious goal of treatment. Given the evidence that deepness and duration of molecular response are necessary but not sufficient requisites for a successful TFR, additional biological criteria are needed to identify CML patients suitable for efficacious discontinuation. Leukemia stem cells (LSCs) are supposed to be the reservoir of the disease. Previously, we demonstrated that residual circulating CD34+/CD38-/CD26+ LSCs were still detectable in a consistent number of CML patients during TFR.

**Methods:** CML LSCs could be easily identified by flow-cytometry as they express the CD34+/CD38-/CD26+ phenotype. In this study, we explored the role of these cells and their correlation with molecular response in a cohort of 109 consecutive chronic phase CML patients prospectively monitored from the time of TKI discontinuation.

**Results:** After a median observation time of 33 months from TKI discontinuation, 38/109 (35%) patients failed TFR after a median time of 4 months, while 71/109 (65%) patients are still in TFR. At TKI discontinuation, peripheral blood CD26+LSCs were undetectable in 48/109 (44%) patients and detectable in 61/109 (56%). No statistically significant correlation between detectable/undetectable CD26+LSCs and the rate of TFR loss was found (*p* = 0.616). The incidence of TFR loss based on the type of TKI treatment was statistically significant for imatinib treatment compared to that of nilotinib (*p* = 0.039). Exploring the behavior of CD26+LSCs during TFR, we observed fluctuating values that were very variable between patients, and they were not predictive of TFR loss.

**Discussion:** Up to date, our results confirm that CD26+LSCs are detectable at the time of TKI discontinuation and during TFR. Moreover, at least for the observation median time of the study, the persistence of “fluctuating” values of residual CD26+LSCs does not hamper the possibility to maintain a stable TFR. On the contrary, even patients discontinuing TKI with undetectable CD26+LSCs could undergo TFR loss. Our results suggest that factors other than residual LSCs “burden” playing an active role in controlling disease recurrence. Additional studies evaluating CD26+LSCs’ ability to modulate the immune system and their interaction in CML patients with very long stable TFR are ongoing.

## Introduction

Chronic myeloid leukemia (CML) is a myeloproliferative neoplasm that is still considered a model disease in cancer for its peculiar biological and clinical characteristics and for having pioneered target therapies and precision medicine approaches (Melo and Barnes, 2007; Minciacchi et al., 2021).

The development of several BCR::ABL1 tyrosine kinase inhibitor (TKI) generations, including imatinib, nilotinib, and dasatinib, currently approved as front-line therapy, revolutionized the dismal prognosis of CML patients, offering a life expectancy similar to that of the general population (Gambacorti-Passerini et al., 2011; Viganò et al., 2014; Bower et al., 2016; Hehlmann et al., 2017; Inzoli et al., 2022). However, side effects, possible correlated toxicity, and not negligible costs of lifelong TKI therapy represent still important issues regarding this disease (Efficace et al., 2011; Williams et al., 2013; Guérin et al., 2014). Today, the expectations rising for CML patients aim both to achieve long-term survival and to stop treatment (Saifullah and Lucas, 2021). It is demonstrated that a fraction of CML patients in sustained deep molecular response (DMR) may discontinue TKI treatment, and about 50% of them will maintain a treatment-free remission (TFR) without recurrence of CML (Bocchia et al., 2018). However, different aspects need to be taken into account regarding CML treatment discontinuation, as overall, just 20% of newly diagnosed CML patients will achieve a successful TFR (Atallah and Sweet, 2021; Chen et al., 2021; Pavlovsky et al., 2021). Attempting TFR is currently recommended for chronic phase (CP) CML patients on TKI treatment for at least 3 years, with a sustained DMR for at least 2 years and without evidence of any ABL kinase domain mutation (Atallah and Sweet, 2021; Pavlovsky et al., 2021). Most studies on TKI discontinuation reported that longer TKI treatment duration is associated with more chance of TFR success (Mahon et al., 2010; Saussele et al., 2018; Fujisawa et al., 2019; Molica et al., 2020; Shah et al., 2020; Richter et al., 2021), and this is strictly linked to DMR duration: CML patients with longer DMR have a lower risk of relapse (Baccarani et al., 2019; Fava et al., 2019; Castagnetti et al., 2021; Iurlo et al., 2022). The preexistence of TKI resistance must be considered, since patients who failed a treatment could also have higher chances of TFR failure, while a prior interferon-alpha (INF-α) treatment, in combination with low-risk factors and TKI therapy, may help to achieve DMR, activating quiescent cells and exposing them to the TKIs’ killing effect (Chen et al., 2021; Puzzolo et al., 2022). The immune system also appears to have a predictive role in higher TFR success since it has been suggested that higher levels of NK cells may be able to eradicate Leukemia Stem Cells (LSCs) and potentiate adaptive immune responses (Hughes and Yong, 2017; Ureshino et al., 2017; Irani et al., 2020; Hsieh et al., 2021). Some studies focused attention on the type of BCR::ABL1 transcript, hypothesizing that the e13a2 transcript has higher risks of molecular relapse compared to e14a2 (Claudiani et al., 2017; Chen et al., 2022). Instead, in terms of prognostic scores, a low-risk Sokal score has better progression-free survival (PFS) and overall survival (OS), with high TFR rate, while the ELTS score could not be predictive of TFR success. Some attempts have also been made to investigate the correlation of TFR success with CML patients’ age, but it is still uncertain why older patients seem to achieve higher chances of TFR compared to younger ones (Breccia et al., 2020; Inzoli et al., 2022). The hypothesis is related to the presence of LSCs, which could be less functional in older patients as for HSCs, thus reducing the risks of relapse (Saifullah and Lucas, 2021).

LSCs are considered the reservoirs of CML; their persistence is correlated to the fact that they are largely quiescent and can resist and escape TKIs’ action using kinase-independent mechanisms (Chen et al., 2021).

Great efforts are ongoing to target these cells, with the aim to eradicate this silent disease fraction and to improve patients’ outcomes (Cea et al., 2013; Shah and Bhatia, 2018; Baquero et al., 2019). In the previous years, several studies, including research conducted by our group, investigated CML-specific LSCs in bone marrow (BM) and peripheral blood (PB) samples (Bocchia et al., 2018). As documented, CML LSCs reside within the CD34+/CD38−/Lin− fraction and are characterized by an aberrant expression of specific surface markers, such as IL1RAP, CD25, CD93, and CD26 (DPPV) (Raspadori et al., 2019). In particular, CD26 (dipeptidylpeptidase IV) is considered a potential biomarker for the identification and quantification of CML LSCs, able to discriminate CML LSCs from normal hematopoietic stem cells from LSCs of other myeloid neoplasms (Galimberti et al., 2018; Sicuranza et al., 2022).

We earlier demonstrated the feasibility of PB CD26+LSC flow cytometry detection in CML patients and documented, in a cross-sectional study, the presence of circulating CML LSCs in a substantial number of patients in DMR both during TKI treatment and during TKI discontinuation (Bocchia et al., 2018). In order to fine-tune and further explore any active role of this staminal CML reservoir, we conducted a multicenter study with the intent to prospectively measure residual CD26+LSCs in CP-CML patients attempting TFR from the time of TKI discontinuation for a minimum of 12 months or until disease relapse, if any.

Specifically, the aims of this study were 1) to evaluate prospectively if a CML LSC “threshold” at the time of discontinuation may have an impact in predicting a successful TFR, 2) to investigate prospectively the behavior of residual LSCs along TFR, and 3) to exploit any correlation between CML LSC persistence, molecular response (MR), and TFR maintenance.

## Materials and methods

### Patient cohort

Consecutive CP-CML patients in sustained DMR meeting standard criteria for attempting TKI discontinuation entered this multicenter study (10 Italian hematology centers involved).

Each enrolled patient was evaluated for PB CD26+LSCs at TKI discontinuation (baseline), at +1, +2, +3, +6, and +12 months of TFR and at disease recurrence. Additionally, +18 and +24 months of TFR have been proposed as optional evaluations.

All centers provided clinical data about disease history, risk scores (EUTOS, SOKAL), cytogenetic alterations, BCR::ABL1 transcript type, line of treatment and duration of TKI therapy, duration of DMR, and MR data for each time point of the study and at the time of molecular relapse. The study was approved by each center’s ethical committee, and all subjects signed an informed consent to participate in the study in accordance with the Declaration of Helsinki and each institution’s policy.

### Flow cytometry analysis

Six milliliters of EDTA PB samples have been collected and centrally analyzed within 24 h at the Siena Flow Cytometry lab. For all subjects, CD26 expression has been evaluated by a standardized multiparametric flow cytometry analysis on CD45+/CD34+/CD38-populations using a four-color staining protocol with the lyse stain wash procedure. Red cell lysis was performed with ammonium chloride (BD Biosciences), 1:10 diluted in deionized water, using the Lyse Wash Assistant instrument (BD Biosciences). After lysis, 2.0 × 10^6^ leukocytes were incubated with a custom-made lyophilized pre-titrated antibody mixture test tube (BD™ Lyotubes, BD) including CD45 V500 (BD Pharmingen clone 2D1), CD34 FITC (BD Pharmingen clone 581), CD38 APC (BD Pharmingen clone HIT2), and CD26 PE (BD Pharmingen clone M-A261) antibodies and a BD stain control tube lacking CD26. To reach a sensitivity of 10^–5^, acquisition and analysis of at least 1.0 × 10^6^ cells was performed in all samples by using the FACSCanto II and FACSLyrics flow cytometer using DIVA 8 and FACSuite software (BD, Biosciences). Additionally, to ensure reproducible results over time, we followed a standardized protocol that implied adjustments of FACS internal parameters, using the CS&T System (BD Biosciences), to keep constant the instrument performance by correcting wear of lasers and fluidic instability. The median absolute number of CD26^+^ cells/μL has been calculated as follows: [(no. WBCs/μL) × (% of CD34+/CD38−/CD26+ on CD45+cells)] (Raspadori et al., 2019).

### Molecular response

BCR::ABL1 breakpoint mRNA monitoring has been assessed locally at each molecular laboratory of the centers participating in the study, by quantitative polymerase chain reaction (qPCR) methods, according to European Leukemia Net (ELN) guidelines (Hochhaus et al., 2020). Molecular response, expressed on an International Scale (IS), has been evaluated at the time of TKI discontinuation and at the subsequent time points (1, 2, 3, 6, 12, 18, and 24 months), until TFR loss, if any.

### Statistical analysis

Descriptive statistics was carried out; qualitative variables were summarized by absolute frequencies and percentages, and the quantitative ones were summarized with median and interquartile range. BCR::ABL1 and CD26+LSCs differences between TFR-sustained and TFR-loss were evaluated with the Mann–Whitney test, and the association between MR or treatment and response was evaluated with the Fisher exact test. The *post hoc* test for the association between treatment and TFR loss was evaluated by multiple Fisher exact tests with Bonferroni correction. Kaplan–Meier curves were performed to evaluate the survival without molecular relapse between patients double positive (i.e., positive for BCR::ABL1 copies and PB residual CD26+LSCs), double negative (i.e., negative for BCR::ABL1 copies and PB residual CD26+LSCs), and discordant at baseline (i.e., one positive and one negative test). A log-rank test was performed to compare the three different groups. A *p* < 0.05 was considered statistically significant. Statistical analyses were carried out with R version 4.0.1.

## Results

### Patient cohort clinical data

Between June 2017 and June 2022, 109 consecutive CP-CML patients at the time of TKI discontinuation were enrolled in the study. Clinical and biological data for CML diagnosis are reported in Table 1. In brief, 62/109 (57%) patients were males, and 47/109 (43%) were females; the median age at diagnosis was 53 years (range 19–76 years). The Sokal score was high in 16/109 (14.5%) patients, intermediate in 38/109 (35%), low in 49/109 (45%), and not available in 6/109 (5.5%), while the EUTOS score was high in 15/109 patients (14%), intermediate in 6/109 (5.5%), low in 82/109 (75%), and unknown in 6/109 (5.5%). The most frequent BCR::ABL1 transcript was b3a2 in 54/109 (49.6%) patients. Additional cytogenetic abnormalities were reported only in 3 patients (2.8%). First-line TKI treatment included imatinib in 39/109 (35.8%) patients, nilotinib in 49/109 (45%), and dasatinib in 21/109 (19.2%). The complete cytogenetic response was achieved at a median time of 3 months (range 2–21 months). Regarding MR after 12 months from TKI start, 56/109 (51.4%) patients had MR ≤ 3, 16/109 (14.7%) had MR 4, 14/109 (12.8%) had MR 4.5, and 16/109 (14.7%) had MR 5. The median TKI treatment duration before TKI withdrawal was 7 years (range 3–18 years). According to TKIs, the median treatment duration before TFR was 8 years (range 4–18 years) for patients on imatinib, 7 years (range 3–17 years) for patients on nilotinib, and 8 years (range 3–15 years) for those on dasatinib. After a median observation time of 33 months (range 2–63 months) from TKI discontinuation, a total of 38/109 (35%) patients failed TFR after a median time of 4 months (range 1–39 months). According to TKI treatment, these 38 TFR failing patients were distributed as follows: 19/39 (48.7%), 11/49 (22.4%), and 8/21 (38.1%) for patients previously treated with imatinib, nilotinib, and dasatinib, respectively.

**TABLE 1:** Clinical data of the whole cohort, patients with TFR loss, and patients with TFR still sustained. WBC: white blood cells; LY: lymphocyte count; TFR: treatment-free remission; MR: molecular response; TKI: tyrosine kinase inhibitor; yrs: years; and mos: months.

Patient’s characteristics	Whole cohort (*n* = 109)	TFR loss (*n* = 38)	TFR sustained (*n* = 71)
Median age at diagnosis (range)	53 years (19–76 years)	51.5 years (27–76 years)	54 years (19–76 years)
Sex			
Male	62 (57%)	20 (53%)	42 (59%)
Female	47 (43%)	18 (47%)	29 (41%)
Median WBC at discontinuation (range)	6000/mmc (3342–12680/mmc)	6000/mmc (3767–12680/mmc)	6040/mmc (3342–9,800/mmc)
Median LY (range)	1680,5/mmc (1000–3465/mmc)	1680,5/mmc (1000–3465/mmc)	1700/mmc (1000–3400/mmc)
Sokal score			
High	16 (14.5%)	4 (10.5%)	12 (17%)
Intermediate	38 (35%)	12 (31.5%)	26 (36.5%)
Low	49 (45%)	20 (53%)	29 (41%)
Unknown	6 (5.5%)	2 (5%)	4 (5.5%)
EUTOS score			
High	15 (14%)	4 (10.5%)	11 (15.5%)
Intermediate	6 (5.5%)	4 (10.5%)	2 (3%)
Low	82 (75%)	28 (74%)	54 (76%)
Unknown	6 (5.5%)	2 (5%)	4 (5.5%)
BCR::ABL1 transcript			
b2a2	27 (24.8%)	11 (28.9%)	16 (22.5%)
b3a2	54 (49.6%)	18 (47.4%)	36 (50.7%)
b2a2/b3a2	7 (6.4%)	2 (5.3%)	5 (7.1%)
b2a2/b3a3	1 (0.9%)	1 (2.6%)	-
Absent	1 (0.9%)	-	1 (1.4%)
Unknown	19 (17.4%)	6 (15.8%)	13 (18.3%)
Additional cytogenetic abnormalities	3 (2.8%)	1 (2.6%)	2 (2.8%)
Median time to complete cytogenetic response (range)	3 months (2–21 months)	3 months (2–21 months)	3 months (2–18 months)
Molecular Response after starting therapy 12 months			
MR ≤ 3	56 (51.4%)	15 (39.5%)	41 (57.7%)
MR = 4	16 (14.7%)	8 (21.1%)	8 (11.3%)
MR = 4,5	14 (12.8%)	8 (21.1%)	6 (8.5%)
MR = 5	16 (14.7%)	4 (10.5%)	12 (16.9%)
Unknown	7 (6.4%)	3 (7.8%)	4 (5.6%)
TKI therapy before discontinuation			
Imatinib	39 (35.8%)	19 (50.0%)	20 (28.2%)
Nilotinib	49 (45%)	11 (28.9%)	38 (53.5%)
Dasatinib	21 (19.2%)	8 (21.1%)	13 (18.3%)
Median TKI treatment duration (range)	7 years (3–18 years)	7 years (7–18 years)	7.5 years (3–17 years)
Median TKI treatment duration according to TKI			
Imatinib	8 years (4–18 years)	8 years (4–18 years)	8 years (4–16 years)
Nilotinib	7 years (3–17 years)	7 years (3–12 years)	7 years (3–17 years)
Dasatinib	8 years (3–15 years)	8 years (4–14 years)	8 years (3–15 years)
Median observation time (range)	33 months (2–63 months)	35 months (4–60 months)	32 months (2–63 months)

### CD26+LSC evaluation and BCR::ABL1 transcript at the time of TKI discontinuation

**FIGURE 1:**
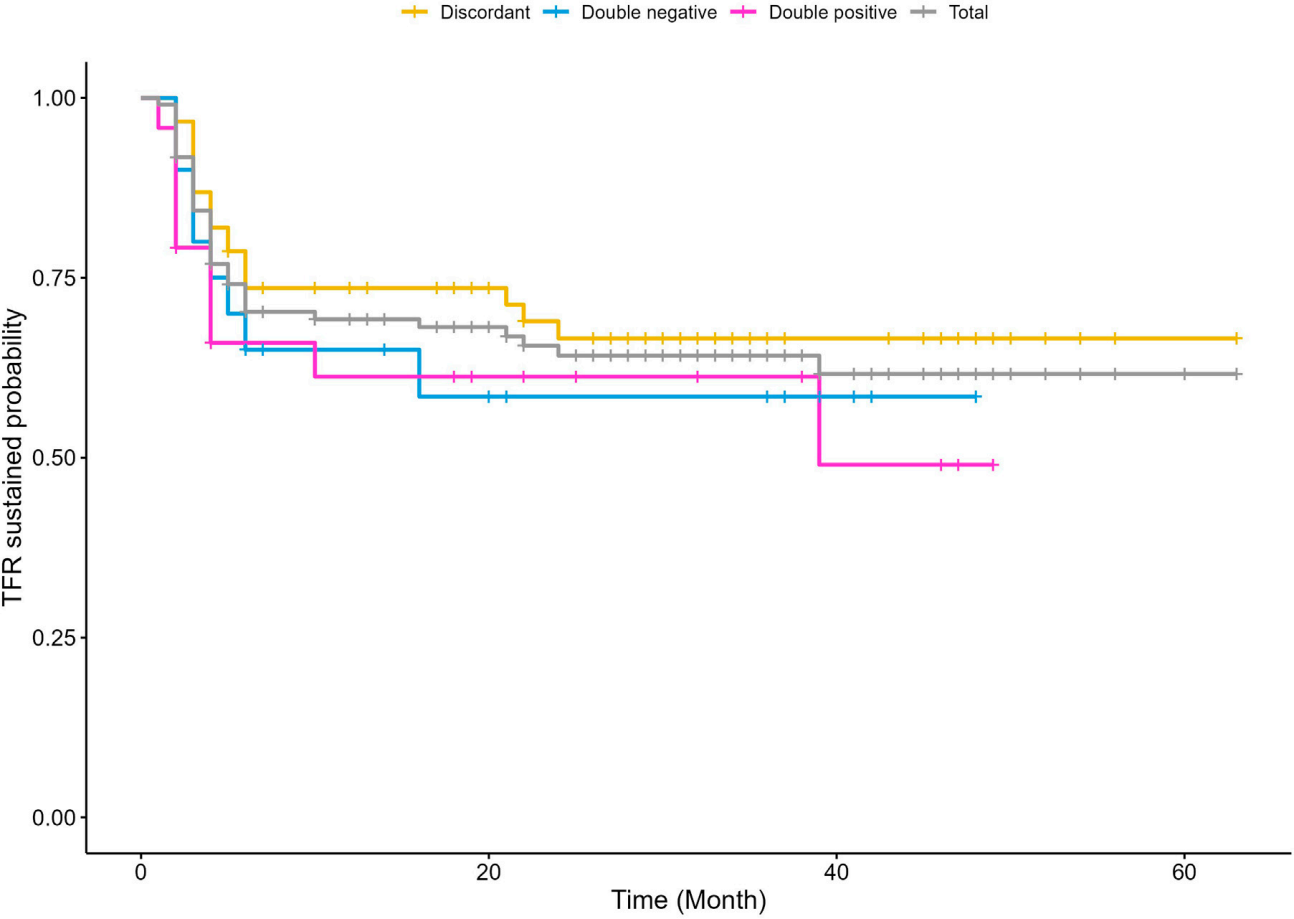
Kaplen–Meier (KM). The survival without molecular relapse is shown in CML patients’ scoring, at the time of TKI discontinuation, “positive” for both CD26+LSCs and BCR::ABL1 copies (i.e., double positive), negative for both CD26+LSCs and BCR::ABL1 copies (i.e., double negative), and “discordant” with either way positive only for CD26+LSCs or BCR::ABL1 (i.e., discordant). In grey is reported the global CML patients’ survival without molecular relapse.

All 109 CP-CML patients were evaluated for PB CD26+LSCs and BCR::ABL1 transcript at the time of TKI discontinuation. In 48/109 (44%), PB CD26+LSCs were undetectable, while in 61/109 (56%), we detected a median of 0,007 CD26^+^ cells/μL (range 0,0001–0,1184 cells/μL). Regarding molecular evaluation at the time of TKI withdrawal, in 57/109 (51%) patients, BCR::ABL1 transcript resulted undetectable, while 52/109 (49%) patients scored positive with a median value of 0,0021 copies (range 0,0002–0,038 copies); in terms of MR, an MR > 3 was documented in 106/109 (97%) patients. No statistically significant correlation between detectable/undetectable CD26+LSCs and the rate of TFR loss was found (*p* = 0.616). Similarly, when correlating BCR::ABL1 ratio or BCR::ABL1 log reduction (expressed as MR4, MR4.5, and MR5) at the time of TKI stop with the incidence of TFR loss, we did not find statistically significant associations (*p* = 0.962 and *p* = 0.275, respectively). Additionally, we also investigated the correlation between molecular relapse and any possible combinations of detectable/undetectable CD26+LSCs and detectable/undetectable BCR::ABL1 transcript without finding statistically significant results (*p* = 0.646). Experimental data are summarized in Table 2; Figure 1. Table 3 details CD26+LSCs and BCR::ABL1 status at the time of imatinib, nilotinib, and dasatinib discontinuation; again, no correlation between residual CD26+LSCs, molecular response, and TFR loss was found. However, the pre-withdrawal type of TKI treatment was associated with TFR loss, and nilotinib treatment was followed by a TFR loss rate that was statistically significantly lower compared to that of imatinib (*p* = 0.039) but not of dasatinib.

**TABLE 2:** Combination between detectable/undetectable CD26+LSC and detectable/undetectable BCR::ABL1 in relapsed/non-relapsed patients at the time of TKI discontinuation. LSCs: leukemia stem cells; TFR: treatment-free remission.

	CD26+LSCs detectable	CD26+LSCs undetectable	CD26+LSCs detectable	CD26+LSCs undetectable
	BCR::ABL1 detectable	BCR::ABL1 undetectable	BCR::ABL1 undetectable	BCR::ABL1 detectable
Patients (*n* = 109)	25/109 (22.9%)	21/109 (19.3%)	36/109 (33.0%)	27/109 (24.7%)
TFR LOSS (*n* = 38)	11/38 (29.0%)	8/38 (21.0%)	12/38 (31.6%)	7/38 (18.4%)
TFR SUSTAINED (*n* = 71)	14/71 (19.7%)	13/71 (18.3%)	24/71 (33.8%)	20/71 (28.2%)

**TABLE 3:** PB CD26+LSCs and BCR::ABL1 transcript at the time of TKI discontinuation, according to treatment. TFR: treatment-free remission.

Type of TKI before discontinuation	Imatinib	Nilotinib	Dasatinib
Patients whole cohort (*n* = 109)	39 (35.8%)	49 (45.0%)	21 (19.2%)
CD26+LSCs at TKI discontinuation			
POSITIVE (range)	19/39 (48.7%) (0,0048–0,1184 cells/μL)	30/49 (61.2%) (0,0001–0,1039 cells/μL)	12/21 (57.1%) (0,0063–0,0695 cells/μL)
NEGATIVE	20 (51.3%)	19 (38.8%)	9 (42.9%)
BCR::ABL1 transcript at TKI discontinuation			
POSITIVE (range)	19/39 (48.7%) (0,001–0,0046 copies)	26/49 (53.1%) (0,00024–0,007 copies)	10/21 (47.6%) (0,001–0,029 copies)
NEGATIVE	20/39 (51.3%)	23/49 (46.9%)	11/21 (52.4%)

### CD26+LSC evaluation during TFR

According to the study design, PB CD26+LSCs were prospectively evaluated during TFR at established time points with a total of 541 measurements. To explore the behavior of residual CD26+LCSs over time, we focused our data analysis on three time points (3, 6, and 12 months) of the 71/109 CML patients still in TFR at the last follow-up.

CD26+LSCs during TFR were undetectable and detectable, respectively, in 24/71 (34%) and 47/71 (66%) patients at 3 months, 27/71(38%) and 44/71 (62%) patients at 6 months, and 24/62 (39%) and 38/62 (61%) patients at 12 months. When detectable, the median values of PB CD26+LSCs were 0,0128, 0,01 and 0,0155 cells/μL at 3, 6, and 12 months, respectively.

**FIGURE 2:**
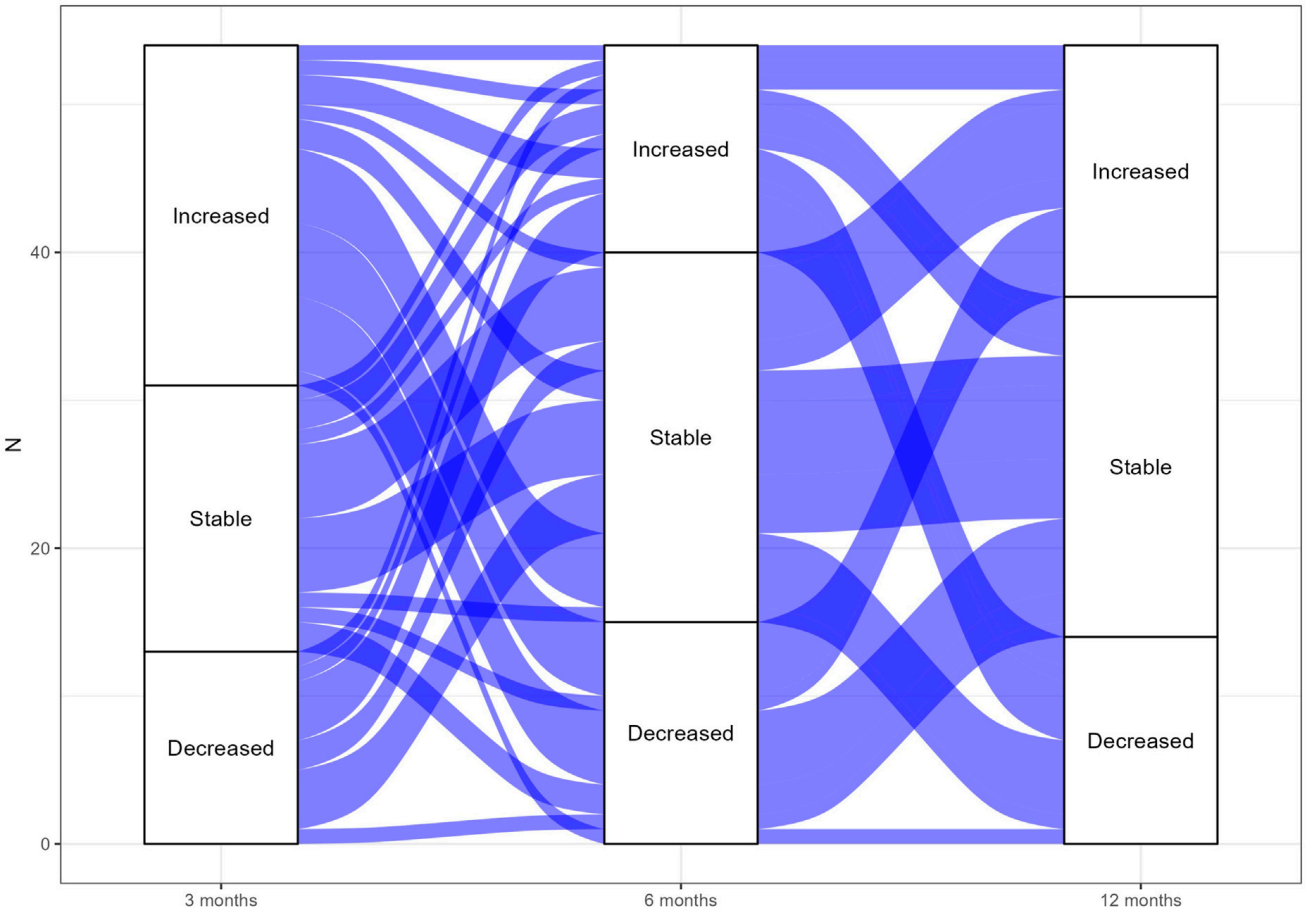
Alluvial plot of CD26+LSCs fluctuations for TFR-sustained patients. The alluvial plot represents CD26+LSCs fluctuations in TFR-sustained patients between 3, 6, and 12 months from TKI stop. The width of the lines corresponds to the number of patients who shared the same behavior.

**FIGURE 3:**
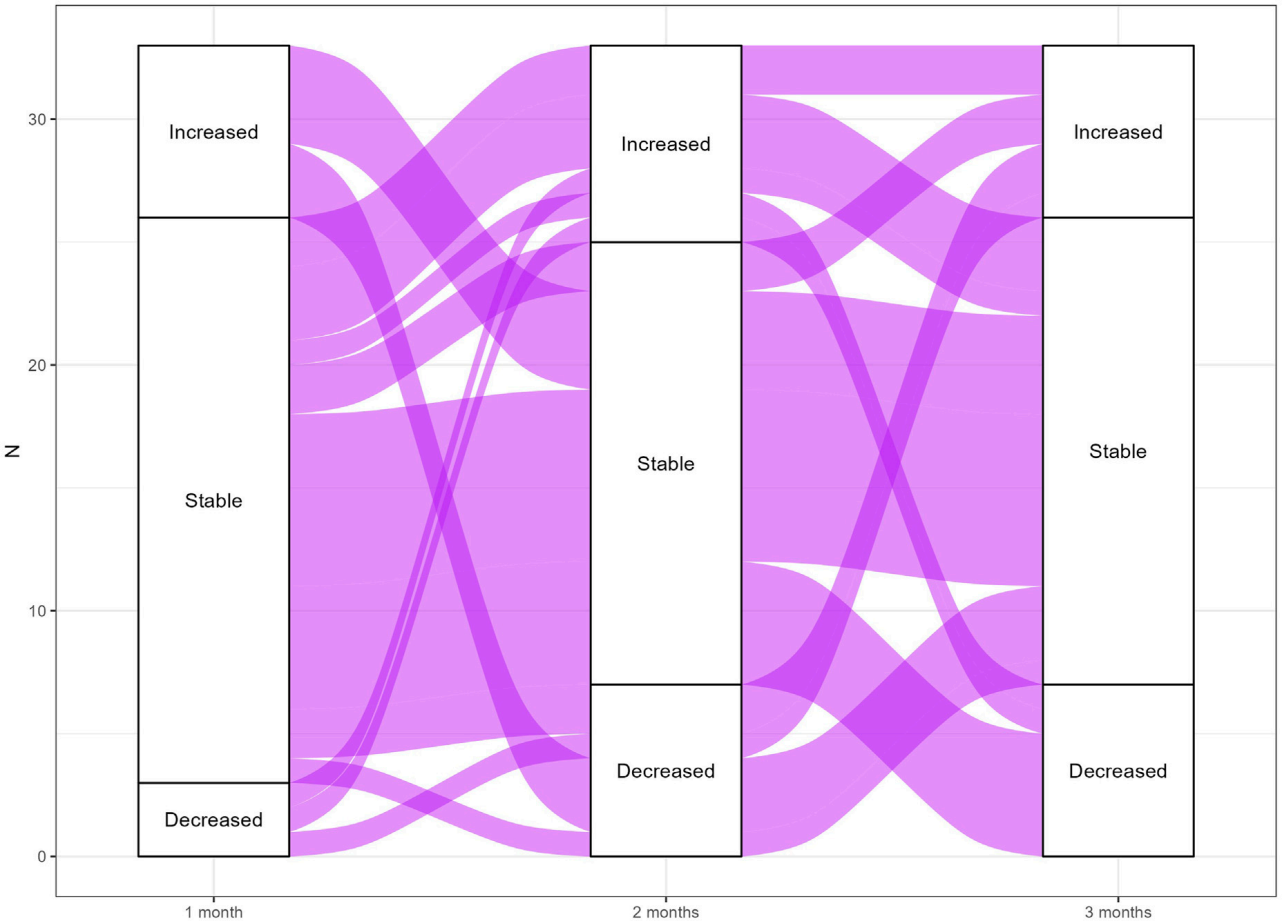
Alluvial plot of CD26+LSCs fluctuations for TFR-loss patients. The alluvial plot represents CD26+LSCs fluctuations in TFR-loss patients between 1, 2, and 3 months from TKI stop. The width of the lines corresponds to the number of patients who shared the same behavior.

**FIGURE 4:**
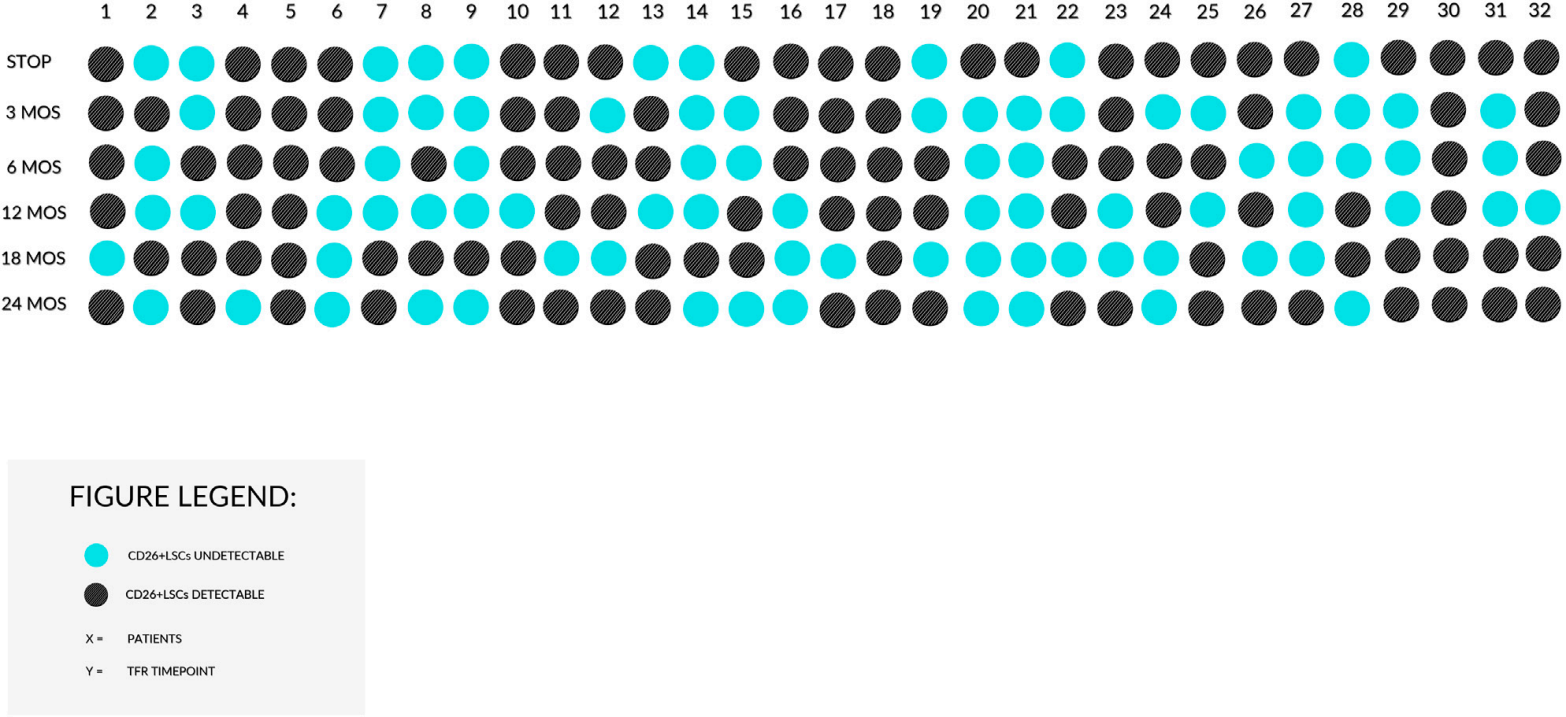
Behavior of CD26+LSCs in 32 TFR patients achieving 24 months of follow-up. The black dots stand for detectable PB CD26+LSCs, and the blue dots stand for undetectable CD26+LSCs. The great variability of the behavior of LSCs among patients with sustained TFR is demonstrated.

Fluctuations of CD26+LSCs were very different between TFR patients and not predictive of TFR loss. To better depict CD26+LSC fluctuations observed during the TFR time, we realized two alluvial plots of TFR-sustained (Figure 2 **)** and TFR-loss patients **(** Figure 3). Furthermore, in a restricted fraction of patients, we analyzed the persistence of residual CD26+LSCs at 18 and 24 months of discontinuation: 25/40 (62.5%) patients at 18 months and 20/32 (62.5%) patients at 24 months of stable TFR still showed detectable CD26+LSCs in their PB with a median number of 0,0096 and 0,0058 cells/μL, respectively. Figure 4 displays CD26+LSCs behavior during TFR in 32 patients that achieved 24 months of follow-up, and it confirms the great variability observed between patients.

## Discussion

Nowadays, in the era of precision and personalized medicine, the achievement of a safe TFR in CML patients discontinuing TKIs is the focal point of hematologists caring for CML patients. Although in the past years, the optimization of TKI use has limited TKI resistance and prompted the achievement of a sustained DMR, a considerable fraction of CML patients attempting TFR soon experience a molecular relapse and need to restart TKI treatment to avoid disease reappearance. The persistence of residual quiescent LSCs post-TKI treatment has been considered responsible for TFR loss. However, the mechanisms involved in this process have not been investigated. To our knowledge, this is the first report that prospectively monitored the behavior of circulating CML-specific CD26+LSCs in CML patients during TFR, exploring their possible active role in inducing molecular relapse. Previously, we demonstrated in a cross-sectional study that a consistent number of CML patients undergoing TKI treatment still harbored measurable residual PB CD26+LSCs, even when displaying a prolonged DMR and during a stable TFR. However, prospective data regarding how residual LSCs would behave over time from TKI withdrawal were lacking. In the prospective multicenter study reported here, 109 CML patients achieving standard criteria for TFR attempt were closely monitored for the presence of residual PB CD26+LSCs together with any clinical or molecular evidence of disease relapse.

From the clinical point of view, in line with the literature, we documented that 35% of CML patients discontinuing TKI treatment failed TFR after a median time of 4 months. We also found that the TKI type of treatment appeared to be correlated to the probability of TFR loss: in particular, imatinib-treated patients showed a significantly higher relapse rate when compared to those treated with nilotinib but not to those with dasatinib.

From the biological point of view, at the time of TKI withdrawal, the majority of CML patients (56%) still harbored a low yet detectable amount of CD26+LSCs in their peripheral blood, without any significant difference according to previous TKI treatment. However, surprisingly, and somehow in contrast to what was expected, the persistence of PB CD26+LSCs at the time of TKI discontinuation did not correlate with the incidence of relapse, and no threshold of residual CD26+LSC number predictive of TFR loss was found. Similarly, no correlation was found between the molecular response (expressed in BCR::ABL1 ratio or expressed in MR) at the time of TFR with the incidence of molecular relapse. Even when we analyzed combined values of PB CD26+LSCs and BCR::ABL1 copies at the time of TKI discontinuation, no statistically significant difference in terms of relapse rate was found between patients resulting in “positive” for both CD26+LSCs and BCR::ABL1 persistence, “negative” (absence of both residual CD26+LSCs and BCR::ABL1 copies), or “discordant” (either way positive for CD26+LSCs or BCR::ABL1).

The second aim of our study was to prospectively investigate the fate of residual CD26+LSCs along TFR and explore their behavior, both in relapsing and non-relapsing CML patients. As reported by alluvial plots (Figure 2 and Figure 3) by measuring CD26+LSCs at several time points during the study core (12 months), we observed fluctuating values of residual LSCs with great variability within each patient and between patients both in the subgroup that lost TFR (Figure 2) and in the 71 CML patients in sustained TFR (Figure 3). The long-lasting persistence of PB CD26+LSCs and the fluctuation of their values was also confirmed in 32 CML patients with stable TFR, in which flow cytometry evaluation of residual LSCs was monitored for up to 24 months (Figure 4).

Taken together, our findings strongly suggest that the persistence of quiescent CD26+LSCs at the time of TKI withdrawal and during TFR does not necessarily result in overt disease recurrence. Nevertheless, all 38 CML patients failing TFR showed clearly detectable PB CD26+LSCs at the time of molecular disease recurrence. The latter raises two questions. Are CD26+LSCs really the expression of a staminal, quiescent fraction of CML? If yes, which factors, if any, control these cells or induce them to enter into proliferation and differentiation pathways, thus causing the reappearance of active disease? To try to answer the first question, we further characterized the phenotype of PB CD26+LSCs and documented the co-expression of specific antigens, such as CD90 and Ki67 (Pacelli et al., 2022). The former confirmed the real stemness property of these CML-specific cell reservoirs; however, the latter, being a marker of “proliferative capability,” is somehow in contrast with the concept of “quiescence” of LSCs. It could then be hypothesized that CD26+LSCs may enter proliferation and differentiation status only under certain conditions, such as if they are able to escape a possible immune control.

Even in the pre-TKI era, like in the last two decades of successful molecularly targeted therapy, several authors focused on both the role of the immune system in concurring with CML control (Caocci et al., 2015; Ilander et al., 2017) and immunotherapeutic strategies to be possibly added to TKIs in order to aim at CML cure (Cayssials and Guilhot, 2017). It seems evident that the unraveling of the inmost features of the TKI-insensitive stem/progenitor cell population, as well as its interactions with the immune system, may introduce an alternative way to monitor the BCR::ABL1-based molecular response to identify CML patients who achieved a cure/immune control of the disease and may, therefore, safely stop TKI. Recently, the PD-1/PD-L1 signaling pathway was described as an adaptive mechanism of immune resistance enacted by tumor cells to evade immune response (Butte et al., 2008). It has been shown that leukemic cells harbor the PD-L1 antigen, although there is little or no information about CML LSCs (Mumprecht et al., 2009; Christiansson et al., 2013; Sehgal et al., 2015).

As such, we have also tried to characterize CML-specific LSCs regarding their possible interaction with the immune system. Preliminary data in 47 newly diagnosed CML patients showed that the co-expression of PD-L1 on their CD26+LSCs is variable: 22/47 (47%) patients scored negative while 25/47 (53%) scored positive, albeit with a quite variable percentage of expression (median 28.5% of CD26^+^ cells, range 12%–82.8%) (Pacelli et al., 2022). These preliminary pieces of evidence are in favor of a possible diverse fate of residual CD26+LSCs in every single patient with regard to immune evasion and, consequently, disease recurrence.

In conclusion, here, we showed prospectively and in a consistent number of CML patients that CML-specific CD26+LSCs may persist at a very low level during TFR, nevertheless allowing patients to live TKI-free and without experiencing a molecular relapse. On the other hand, the possibility to fail TFR is possible even when no CD26+LSCs are detectable at the time of TKI discontinuation. Other factors, including a variable interaction with the host immune system, may be crucial to unravel the fate of these cells and, consequently, to understand if and how to try to eradicate them with alternative approaches. Additional studies exploring the host immune system and its interaction with residual CD26+LSCs in CML patients with very long stable TFR are ongoing.

## Data Availability

The original contributions presented in the study are included in the article/Supplementary Material; further inquiries can be directed to the corresponding authors.
